# Associations of foot ulceration with quality of life and psychosocial determinants among patients with diabetes; a case-control study

**DOI:** 10.1186/s13047-019-0367-5

**Published:** 2019-12-10

**Authors:** Fahad D. Alosaimi, Reem Labani, Nouf Almasoud, Nora Alhelali, Lamya Althawadi, Dhaherah Mani AlJahani

**Affiliations:** 1 Department of Psychiatry, King Saud University, Riyadh, Saudi Arabia; 2 Department of Psychiatry, College of medicine, King Saud University, P.O. Box: 7805, Riyadh, Postcode:11472 Saudi Arabia; 3 College of Medicine, King Saud University, Riyadh, Saudi Arabia

**Keywords:** Diabetic foot, Quality of life, Anxiety, Depression, Saudi Arabia

## Abstract

**Background:**

Patients with diabetic foot ulcers may have a lower quality of life. The objective was to compare the quality of life and its psychosocial determinants among patients with and without diabetic foot ulcers.

**Methods:**

A case-control study was conducted in 2017 among patients with (cases) and without (controls) diabetic foot ulcers. The study tools included the World Health Organization’s Quality of Life scale (WHOQOL-BREF), the Hospital Anxiety and Depression (HAD) scale for anxiety and depression, the Patient Health Questionnaire Physical Symptoms (PHQ-15) for the severity of somatic symptoms, and the Summary of Diabetes Self-Care Activities (SDSCA) measure for self-management.

**Results:**

A total of 209 patients (45 cases and 164 controls) were included. The average age was 56.2 ± 11.7 years, and 55.5% were female. The average scores of WHOQOL-BREF, PHQ-15, and SDSCA were 74.4% ± 12.1% and 8.1 ± 6.1, and 30.4 ± 21.8, respectively. The prevalence of anxiety and depression were 19.6 and 24.9%, respectively. SDSCA was the only psychosocial determinants higher in cases than controls (mean difference = 15.0, 95% CI = -8.0–22.0). The correlation coefficients of WHOQOL-BREF scores with anxiety, depression, and PHQ-15 scores in all patients were − 0.559 (*p* < 0.001), − 0.582 (*p* < 0.001), and − 0.532 (*p* < 0.001), respectively, with similar numbers in both groups. In multivariate analysis, only the association between quality of life and depression was maintained.

**Conclusion:**

Quality of life and psychosocial determinants with the exception of self-management were not associated with diabetic foot ulcers. Depressive symptoms were independent determinant of poor quality of life, irrespective of the status of diabetic foot ulcers.

## Background

According to recent reports of the International Diabetes Federation, the prevalence of diabetes in Saudi Arabia was estimated at 18.5%, which is considered one of the highest in the world [1]. In 2017, it was estimated that approximately 3.8 million patients had diabetes in Saudi Arabia, with 15,000 diabetes-related deaths annually [1]. Diabetic foot disease is a common chronic diabetic complication and typically presents as ulcers with or without infection, in the presence of peripheral neuropathy or peripheral vascular disease [2]. Diabetic foot ulcers affect approximately 8.5% (range 1.8–19%) of the patients with diabetes in Saudi Arabia [3]. This is higher than global prevalence that has been estimated at 6.3% (95% confidence interval, 5.4–7.3%) [4]. Self-management including specific foot-care practices has been recognized as an important preventive measure for diabetic foot ulcers [5, 6].

Diabetic foot ulcers can lead to a significant increase in mortality and lower limb amputations [7, 8]. According to the Saudi National Diabetes Registry, diabetic foot ulcers increase the mortality of diabetic patients by almost two-fold [9]. Additionally, diabetic foot ulcers increase hospital admissions and emergency visits, representing a significant financial burden for patients and healthcare system [10, 11]. Furthermore, diabetic foot ulcers, which are frequently associated with pain and discomfort, have been shown to negatively impact various domains of quality of life in both international [12, 13] and local studies [14, 15]. This association was observed especially in patients who underwent amputations [11] and may be related to the impacts of limited physical functioning, psychological problems, financial burden, and poor social situations [13, 16]. Previous studies showed a link between anxiety/depression and poor quality of life among patients with diabetic foot ulcers [17, 18].

Unlike the traditional risk factors of diabetic foot ulcers in Saudi Arabia [19, 20], psychosocial determinants [21] and quality of life [14, 15] in these patients have received little attention. Additionally, there is a lack of studies examining psychosocial and other related determinants of quality of life. The objective of the current study was to compare the quality of life and its psychosocial determinants (anxiety, depression, severity of somatic symptoms, and self-management) among patients with and without diabetic foot ulcers. A secondary objective was to assess the associations of these psychosocial determinants with the quality of life.

## Methods

### Setting

The current study was conducted at the diabetic center and the primary care clinic of King Saud University Medical City in Riyadh (KSUMC), Saudi Arabia. KSUMC is a 1000-bed tertiary care medical teaching facility affiliated with King Saud University. KSUMC is governmentally funded and provides free primary to tertiary healthcare services for primarily patients living in the Riyadh region.

### Study design

A case-control study was conducted between March and August 2017. Patients who were diagnosed with diabetic foot ulcers at the time of study were considered cases. Patients who were not diagnosed with diabetic foot ulcers at the time of study (other than painful peripheral neuropathy) were considered controls. Diabetic foot ulcers were defined as per the International Working Group on the Diabetic Foot; breaks in the skin of the foot in a patient with diabetes, commonly associated with infection, neuropathy and/or peripheral arterial disease [2]. Cases and controls were group-matched by age and sex. Ethical approval was acquired from the institutional review board at the Faculty of Medicine at King Saud University in Riyadh, Saudi Arabia.

### Population

The target population was conveniently recruited from adult patients with diabetes receiving diabetic care at the diabetic center or the primary care clinic of KSUMC. Patients with serious late complications of diabetes, such as dialysis and blindness, were excluded. Patients with difficulty communicating, such as those with a language barrier or severe cognitive impairment, were excluded. There were no exclusions based on the type of diabetes, comorbidities, or glycemic control.

### Sample size

The quality of life score was assumed to be lower in patients with diabetic foot ulcers than in those without diabetic foot ulcers. As the prevalence of diabetic foot ulcers was small, 4 controls were chosen for each case. It was estimated that 40 cases and 160 controls would be required to detect a 10% difference between the two groups (50% ± 20% versus 60% ± 20%), based on 80% power and 95% significance levels. OpenEpi Version 2.2 (http://www.openepi.com) was used for sample size calculation.

### Instruments

A structured study questionnaire that was developed by the study investigators was used to collect the patient information. The information collected included sociodemographic characteristics (Table 1), clinical history (Table 2), and psychological history (top part of Table 3). Additionally, patients’ records were reviewed to detect comorbidities. Standardized tools were used to collect data on the study outcomes. These tools included the World Health Organization’s Quality of Life Instrument-short version (WHOQOL-BREF) for quality of life [22], the Hospital Anxiety and Depression (HAD) scale for the screening of anxiety and depression [23], the Patient Health Questionnaire Physical Symptoms (PHQ-15) for the severity of somatic symptoms [24], and the Summary of Diabetes Self-Care Activities (SDSCA) measure for diabetes self-management [25].

**Table 1:** Demographic characteristics of 209 Saudi diabetic patients by diabetic foot ulcer status^a^

	Diabetic foot ulcers		Total (*N* = 209)	Magnitude of difference ^b^	*p*-value
	Yes (*n* = 45)	No (*n* = 164)			
Age (years)					
Mean ± SD	56.7 ± 12.3	56.1 ± 11.6	56.2 ± 11.7	0.7 (−3.3–4.6)	0.740
< 60	24 (54.5%)	99 (61.5%)	123 (60.0%)	–	0.405
≥ 60	20 (45.5%)	62 (38.5%)	82 (40.0%)	1.36 (0.69–2.66)	
Weight (kg)	88.8 ± 20.4	82.2 ± 20.2	83.6 ± 20.4	6.6 (−1.1–14.3)	0.091
Height (cm)	165.4 ± 8.7	161.8 ± 8.8	162.6 ± 8.9	3.6 (0.2–7.0)	0.037
Body mass index					
Mean ± SD	32.7 ± 7.8	31.7 ± 7.6	31.9 ± 7.6	1.0 (−2.0–4.0)	0.509
Normal	4 (12.1%)	19 (16.5%)	23 (15.5%)	–	0.743
Overweight	11 (33.3%)	32 (27.8%)	43 (29.1%)	1.72 (0.48–6.14)	
Obese	18 (54.5%)	64 (55.7%)	82 (55.4%)	1.41 (0.43–4.64)	
Gender					
Male	23 (51.1%)	70 (42.7%)	93 (44.5%)	1.43 (0.74–2.78)	0.314
Female	22 (48.9%)	94 (57.3%)	116 (55.5%)	–	
Marital status					
Single	3 (6.7%)	7 (4.3%)	10 (4.8%)	–	0.197
Married	33 (73.3%)	132 (80.5%)	165 (78.9%)	0.75 (0.19–2.93)	
Widow	5 (11.1%)	21 (12.8%)	26 (12.4%)	0.71 (0.14–3.65)	
Divorced	4 (8.9%)	4 (2.4%)	8 (3.8%)	3.0 (0.45–20.15)	
Educational level					
Illiterate	7 (16.3%)	34 (21.4%)	41 (20.3%)	–	0.407
Less than high school	17 (39.5%)	43 (27.0%)	60 (29.7%)	1.92 (0.72–5.16)	
High school	10 (23.3%)	37 (23.3%)	47 (23.3%)	1.31 (0.45–3.84)	
University or above	9 (20.9%)	45 (28.3%)	54 (26.7%)	0.97 (0.33–2.87)	
Occupation					
Field job	3 (6.7%)	15 (9.1%)	18 (8.6%)	0.79 (0.21–3.02)	0.651
Office job	9 (20.0%)	21 (12.8%)	30 (14.4%)	1.69 (0.66–4.31)	
Housewife	15 (33.3%)	57 (34.8%)	72 (34.4%)	1.04 (0.48–2.24)	
Not working	18 (40.0%)	71 (43.3%)	89 (42.6%)	–	
Monthly family income (SR)					
< 5000	20 (47.6%)	55 (36.2%)	75 (38.7%)	–	0.172
5000-9999	12 (28.6%)	48 (31.6%)	60 (30.9%)	0.69 (0.31–1.55)	
10,000-14,999	8 (19.0%)	23 (15.1%)	31 (16.0%)	0.96 (0.37–2.48)	
≥ 15,000	2 (4.8%)	26 (17.1%)	28 (14.4%)	0.21 (0.05–0.97)	
Number of family members					
Mean ± SD	6.8 ± 3.1	7.0 ± 3.4	7.0 ± 3.3	−0.2 (−1.4–0.9)	0.718
Living alone					
No	39 (86.7%)	143 (88.3%)	182 (87.9%)	–	0.770
Yes	6 (13.3%)	19 (11.7%)	25 (12.1%)	1.16 (0.43–3.10)	

^a^ Number (frequency) unless noted otherwise; *SD* standard deviation. ^b^ Magnitude of difference between cases and control; odds ratio (for categorical data) or mean difference (for continuous data) plus 95% confidence interval. Empty cells means reference group

**Table 2:** Clinical characteristics of 209 Saudi diabetic patients by diabetic foot ulcer status^a^

	Diabetic foot ulcers		Total (*N* = 209)	Magnitude of difference ^b^	*p*-value
	Yes (*n* = 45)	No (*n* = 164)			
Type of diabetes					
Type 1	4 (8.9%)	33 (20.1%)	37 (17.7%)	0.21 (0.06–0.75)	0.033
Type 2	29 (64.4%)	110 (67.1%)	139 (66.5%)	0.46 (0.20–1.05)	
Unknown	12 (26.7%)	21 (12.8%)	33 (15.8%)	–	
HbA1c					
Mean ± SD	9.9 ± 2.7	8.4 ± 2.0	8.6 ± 2.2	1.5 (0.3–2.7)	0.016
≥ 8	18 (78.3%)	53 (54.1%)	71 (58.7%)	3.06 (1.05–8.89)	0.034
< 8	5 (21.7%)	45 (45.9%)	50 (41.3%)	–	
Age at diagnosis of diabetes	37.6 ± 12.0	42.7 ± 12.0	41.6 ± 12.1	−5.1 (−9.1--1.1)	0.014
Family history of diabetes					
No	14 (31.1%)	46 (28.0%)	60 (28.7%)	–	0.688
Yes	31 (68.9%)	118 (72.0%)	149 (71.3%)	0.86 (0.42–1.77)	
Symptoms					
Peripheral tingling	23 (52.3%)	68 (42.5%)	91 (44.6%)	1.48 (0.76–2.89)	0.248
Peripheral numbness	35 (79.5%)	78 (48.8%)	113 (55.4%)	4.09 (1.85–9.06)	< 0.001
Peripheral hypo- or hyperthermia	20 (45.5%)	70 (43.8%)	90 (44.1%)	1.07 (0.55–2.10)	0.840
Peripheral painlessness	17 (38.6%)	16 (10.0%)	33 (16.2%)	5.67 (2.55–12.57)	< 0.001
Peripheral edema	10 (22.7%)	39 (24.4%)	49 (24.0%)	0.91 (0.41–2.02)	0.821
Tiredness without working	15 (34.1%)	80 (50.0%)	95 (46.6%)	0.52 (0.26–1.04)	0.061
Blurred vision	20 (45.5%)	54 (33.8%)	74 (36.3%)	1.64 (0.83–3.22)	0.153
Nocturnal dyspnea	4 (9.1%)	27 (16.9%)	31 (15.2%)	0.49 (0.16–1.49)	0.203
Loss of concentration	13 (29.5%)	35 (21.9%)	48 (23.5%)	1.50 (0.71–3.17)	0.288
Amputation	10 (22.7%)	0 (0.0%)	10 (4.9%)	NA	< 0.001
Number of comorbid diseases					
Mean ± SD	2.9 ± 1.9	2.8 ± 2.0	2.8 ± 2.0	0.1 (−0.5–0.8)	0.702
None	5 (11.1%)	19 (11.7%)	24 (11.5%)	–	0.715
One or two	16 (35.6%)	68 (41.7%)	84 (40.4%)	0.89 (0.29–2.76)	
Three or more	24 (53.3%)	76 (46.6%)	100 (48.1%)	1.20 (0.41–3.56)	
Comorbidity					
Hypercholesterolemia	20 (44.4%)	97 (59.5%)	117 (56.3%)	0.54 (0.28–1.06)	0.071
Hypertension	28 (62.2%)	76 (46.6%)	104 (50.0%)	1.89 (0.96–3.71)	0.064
Joint diseases	10 (22.2%)	61 (37.4%)	71 (34.1%)	0.48 (0.22–1.03)	0.057
Cataracts	11 (24.4%)	32 (19.6%)	43 (20.7%)	1.32 (0.61–2.90)	0.480
Cardiovascular diseases	12 (26.7%)	29 (17.8%)	41 (19.7%)	1.68 (0.78–3.64)	0.185
Retinal disease	14 (31.1%)	20 (12.3%)	34 (16.3%)	3.23 (1.47–7.08)	0.002
Gastrointestinal disease	5 (11.1%)	26 (16.0%)	31 (14.9%)	0.66 (0.24–1.83)	0.420
Lung disease	5 (11.1%)	24 (14.7%)	29 (13.9%)	0.72 (0.26–2.02)	0.536
Anemia	4 (8.9%)	25 (15.3%)	29 (13.9%)	0.54 (0.18–1.64)	0.269
Renal disease	4 (8.9%)	15 (9.2%)	19 (9.1%)	0.96 (0.30–3.06)	> 0.99
Others	14 (31.1%)	34 (20.9%)	48 (23.1%)	2.14 (0.68–6.74)	0.148

^a^ Number (frequency) unless noted otherwise; *SD* standard deviation. ^b^ Magnitude of difference between cases and control; odds ratio (for categorical data) or mean difference (for continuous data) plus 95% confidence interval. Empty cells means reference group; NA, difference cannot be calculated

**Table 3:** Psychological characteristics and psychosocial examination results of 209 Saudi diabetic patients by diabetic foot ulcer status^a^

	Diabetic foot ulcers		Total (*N* = 209)	Magnitude of difference ^b^	*p*-value
	Yes (*n* = 45)	No (*n* = 164)			
Family history of psychiatric illness					
No	36 (90.0%)	141 (88.1%)	177 (88.5%)	–	> 0.99
Yes	4 (10.0%)	19 (11.9%)	23 (11.5%)	0.83 (0.26–2.58)	
Visit to psychiatrist					
No	33 (75.0%)	149 (90.9%)	182 (87.5%)	–	0.005
Yes	11 (25.0%)	15 (9.1%)	26 (12.5%)	3.31 (1.40–7.86)	
Psychological/behavioral therapy					
No	33 (82.5%)	149 (93.1%)	182 (91.0%)	–	0.058
Yes	7 (17.5%)	11 (6.9%)	18 (9.0%)	2.87 (1.04–7.97)	
Psychiatric medications					
No	30 (75.0%)	148 (93.1%)	178 (89.4%)	–	0.002
Yes	10 (25.0%)	11 (6.9%)	21 (10.6%)	4.49 (1.75–11.50)	
Social support from family or friends					
No	10 (25.0%)	33 (20.8%)	43 (21.6%)	–	0.062
Yes	29 (72.5%)	99 (62.3%)	128 (64.3%)	0.97 (0.43–2.19)	
Somewhat	1 (2.5%)	27 (17.0%)	28 (14.1%)	0.12 (0.02–1.02)	
Psychological deterioration after diagnosis of diabetes					
No	21 (52.5%)	93 (58.1%)	114 (57.0%)	–	0.771
Yes	10 (25.0%)	38 (23.8%)	48 (24.0%)	1.17 (0.50–2.71)	
Somewhat	9 (22.5%)	29 (18.1%)	38 (19.0%)	1.37 (0.57–3.33)	
Improved family relationship after diagnosis of diabetes					
No	9 (22.5%)	54 (33.8%)	63 (31.5%)	–	0.216
Yes	23 (57.5%)	68 (42.5%)	91 (45.5%)	2.03 (0.87–4.75)	
Somewhat	8 (20.0%)	38 (23.8%)	46 (23.0%)	1.26 (0.45–3.57)	
Quality of life; WHOQOL-BREF (mean ± SD)					
Domain 1: Physical health	67.1 ± 19.2	71.1 ± 16.4	70.2 ± 17.1	−4.0 (−9.7–1.7)	0.263
Domain 2: Psychological	75.4 ± 14.9	78.0 ± 13.3	77.4 ± 13.7	−2.6 (−7.1–2.0)	0.384
Domain 3: Social relationships	77.3 ± 16.5	77.5 ± 17.5	77.4 ± 17.3	−0.2 (−5.9–5.5)	0.712
Domain 4: Environment	75.1 ± 16.7	74.7 ± 13.8	74.8 ± 14.5	0.4 (−4.4–5.2)	0.672
Overall quality of life and general health	75.6 ± 13.2	75.1 ± 17.2	75.2 ± 16.4	0.4 (−5.0–5.9)	0.891
Total: All above domains	73.2 ± 12.4	74.8 ± 12.0	74.4 ± 12.1	−1.5 (− 5.5–2.5)	0.539
Anxiety; HAD anxiety					
Mean ± SD	4.7 ± 4.6	4.1 ± 4.4	4.2 ± 4.5	0.6 (−0.9–2.1)	0.355
Normal 0–7	34 (75.6%)	134 (81.7%)	168 (80.4%)	–	0.357
Anxiety > 7	11 (24.4%)	30 (18.3%)	41 (19.6%)	1.45 (0.66–3.17)	
Depression; HAD depression					
Mean ± SD	5.7 ± 4.3	4.7 ± 3.7	4.9 ± 3.9	1.0 (−0.3–2.3)	0.200
Normal 0–7	30 (66.7%)	127 (77.4%)	157 (75.1%)	–	0.139
Depression > 7	15 (33.3%)	37 (22.6%)	52 (24.9%)	1.72 (0.84–3.53)	
Somatic symptoms; PHQ-15					
Mean ± SD	6.8 ± 5.7	8.5 ± 6.2	8.1 ± 6.1	−1.6 (−3.7–0.4)	0.114
Minimal severity (0–4)	18 (40.0%)	59 (36.0%)	77 (36.8%)	–	0.213
Low severity (5–9)	16 (35.6%)	40 (24.4%)	56 (26.8%)	1.31 (0.60–2.87)	
Medium severity (10–14)	7 (15.6%)	33 (20.1%)	40 (19.1%)	0.70 (0.26–1.84)	
High severity (≥15)	4 (8.9%)	32 (19.5%)	36 (17.2%)	0.41 (0.13–1.32)	
Self-Management; SDSCA (mean ± SD)					
Diet	44.8 ± 39.3	29.2 ± 36.9	32.6 ± 37.9	15.5 (3.1–28.0)	0.011
Exercise	17.1 ± 26.1	18.7 ± 27.2	18.4 ± 26.9	−1.6 (−10.5–7.4)	0.351
Blood sugar testing	55.4 ± 37.9	36.1 ± 37.2	40.2 ± 38.1	19.3 (6.9–31.7)	0.003
Foot care	50.8 ± 35.7	23.4 ± 33.7	29.2 ± 35.9	27.4 (16.0–38.8)	< 0.001
Total	42.2 ± 20.3	27.2 ± 21.2	30.4 ± 21.8	15.0 (8.0–22.0)	< 0.001

^a^ Number (frequency) unless noted otherwise. Abbreviations: *SD* standard deviation, *WHOQOL-BREF* World Health Organization Quality of Life Assessment, *HAD* Hospital Anxiety and Depression scale, *PHQ-15* the Patient Health Questionnaire Physical Symptoms; *SDSCA* Summary of Diabetes Self-Care Activities. ^b^ Magnitude of difference between cases and control; odds ratio (for categorical data) or mean difference (for continuous data) plus 95% confidence interval. Empty cells means reference group

### Instrument validation

The study questionnaire was (face and content) validated by experts in psychiatry, diabetes and epidemiology. A pilot study was then conducted with 20 participants, and the questionnaire was slightly modified based on feedback from the pilot study. Validated Arabic versions of the standardized tools mentioned above were used [26–29]. These tools included WHOQOL-BREF [29], HAD [26], PHQ-15 [28], and SDSCA [27].

### Outcome evaluation

The 26 items of WHOQOL-BREF were grouped into 4 domains: physical health, psychological health, social relationships, and environment [30]. Individual and total WHOQOL-BREF scores were transformed into a 100-point scale, with higher scores indicating better quality of life. HAD scores for depression and anxiety were calculated from 7 questions each, with a range between 0 and 21 points. Depression and anxiety were indicated by HAD scores above 7 [31]. The PHQ-15 score was calculated from 15 questions, with a range between 0 and 30. Using the PHQ-15 score, the severity of somatic symptoms was categorized as minimal (0–4), low (5–9), medium (10–14), and high (≥15) [24]. The 8 items of the SDSCA were grouped into 4 groups: diet, exercise, blood sugar testing, and foot care in diabetic patients. As there are no cutoff points, the average SDSCA scores were presented to indicate the frequency of proper self-management of diabetes during the last week [25].

### Statistical analysis

Data are presented as frequencies and percentages for categorical data and the mean and standard deviation (SD) for continuous data. For the first objective, the quality of life and its possible demographic, clinical, and psychological determinants were compared between those with and without diabetic foot ulcer, using the chi-square test or Fisher’s exact test (as appropriate) for categorical data and Student’s t-test or the Mann-Whitney U Test (as appropriate) for continuous data. For the second objective, correlations between quality of life and other psychosocial determinants such as anxiety and depression were examined using the Spearman correlation coefficient. To identify which of these psychosocial determinants were independent determinants of quality of life, a multivariate general linear regression model was run after adjusting for demographic and clinical characteristics (as described in the footnote of Table 4). The outcome was the quality of life, the grouping factor was diabetic foot ulcers, and the predictors of interest were psychosocial determinants; anxiety, depression, somatic symptoms, and self-management (Table 4). All *P*-values were two-tailed. A *P*-value < 0.05 was considered significant. SPSS software (release 25.0, Armonk, NY: IBM Corp) was used for statistical analysis.

**Table 4:** Multivariate linear regression for the determinants of quality of life among 209 Saudi diabetic patients

	Unstandardized beta	Standard error of beta	95% confidence interval (CI)		*p*-value
			Lower CI	Upper CI	
Anxiety; HAD anxiety	−0.034	0.029	−0.094	0.025	0.251
Depression; HAD depression	−0.069	0.030	−0.129	−0.009	0.026
Somatic symptoms; PHQ-15	−0.046	0.037	−0.120	0.029	0.224
Self-management; SDSCA	0.036	0.032	−0.029	0.102	0.270
Diabetic foot ulcer	0.989	1.059	0.882	1.108	0.848

Abbreviations as listed in Table 3

Adjusted for age, gender, BMI, marital status, educational level, occupation, monthly income, number of family members, HBA1c level, and number of comorbid diseases

## Results

### Demographic characteristics by diabetic foot ulcer group

A total of 209 patients (45 cases and 164 controls) were included in the current analysis. The demographic characteristics of the study patients are shown in Table 1. The average age was 56.2 ± 11.7 years, and 40.0% of the patients were 60 years or older. Approximately 55.5% were female. The average body mass index (BMI) was 31.9 ± 7.6, and 55.4% of the patients were considered obese. The majority (78.9%) of patients were currently married. The patients had a wide range of educational backgrounds; 20.3% were illiterate, 29.7% had a less than high school level of education, 23.3% had a high school education, and 26.7% had a university education or above. The majority of patients were either unemployed (42.6%) or housewives (34.4%). The majority of the patients (69.6%) had a monthly income less than SR 10,000. The average family size was 7.0 ± 3.3, and the majority of the patients (87.9%) lived with their families. None of the above demographic characteristics were significantly different between cases and controls.

### Clinical characteristics by diabetic foot ulcer group

The clinical characteristics of the examined patients are shown in Table 2. The majority (66.5%) of the patients had type 2 diabetes. The average level of glycated hemoglobin (HBA1c) was 8.6 ± 2.2, and only 41.3% of the patients had controlled HBA1c (< 8). The average age at diabetes diagnosis was 41.6 ± 12.1 years. The majority (71.3%) had a family history of diabetes. The most frequent symptoms were peripheral numbness (55.4%), tiredness without working (46.6%), peripheral tingling (44.6%), and peripheral hypo- or hyperthermia (44.1%). The average number of comorbid diseases was 2.8 ± 2.0, and 11.5% of patients had no comorbid diseases and 48.1% had three or more diseases. The most frequent comorbid diseases were hypercholesterolemia (56.3%), hypertension (50.0%), joint disease (34.1%), cataracts (20.7%), and cardiovascular diseases (19.7%). Compared with controls, cases had a younger age at diabetes diagnosis (37.6 ± 12.0 versus 42.7 ± 12.0, *p* = 0.014), were less likely to have type 1 diabetes (8.9% versus 20.1%, *p* = 0.033) or to control their diabetes (21.7% versus 45.9%, *p* = 0.034), and were more likely to have peripheral numbness (79.5% versus 48.8%, *p* < 0.001), peripheral painlessness (38.6% versus 10.0%, *p* < 0.001), and retinal disease (31.1% versus 12.3%, *p* = 0.002). Amputation was reported only among those with diabetic foot ulcers (22.7%).

### Psychological determinants by diabetic foot ulcer group

Psychological characteristics and psychosocial examinations of the examined patients are shown in Table 3. Compared with controls, cases were more likely to visit a psychiatrist (25.0% versus 9.1%, *p* = 0.005) and to take psychiatric medications (25.0% versus 6.9%, p = 0.002). There were no significant differences between cases and controls regarding family history of psychiatric illness, receiving psychological/behavioral therapy, social support from family or friends, psychological deterioration after diagnosis of diabetes, or the status of family relationships after diagnosis of diabetes. The average overall WHOQOL-BREF score was 74.4 ± 12.1%, with no differences between cases and controls in individual or overall scores. Approximately 19.6% of the patients had anxiety and 24.9% had depression, with no differences between cases and controls. Out of a maximum of 30, the average PHQ-15 score was 8.1 ± 6.1, with one-third of the patients having either moderate (19.1%) or severe (17.2%) somatic symptoms. The severity of somatic symptoms was slightly lower in cases than in controls, but the differences were not significant. The overall frequency of proper self-management of diabetes was significantly higher in cases than in controls (42.2 ± 20.3 versus 27.2 ± 21.2, *p* < 0.001). This finding was true for all components of self-management, with the exception of physical activity.

### The correlations of quality of life with psychosocial determinants

The correlations of quality of life with psychosocial determinants and comorbidities are shown in Table 5. There were significant strong negative correlations between quality of life and anxiety, depression, and the severity of somatic symptoms. For example, the correlation coefficients of the correlations of the total WHOQOL-BREF score (all domains) with the HAD anxiety, HAD depression, and PHQ-15 scores in all patients were − 0.559 (*p* < 0.001), − 0.582 (*p* < 0.001), and − 0.532 (*p* < 0.001), respectively. These numbers were very similar in cases and controls. On the other hand, some domains of quality of life had significant mild to moderate positive correlations with diet and exercise in both cases and controls. For example, the overall WHOQOL-BREF score in cases correlated with the scores on the diet and exercise components of the SDSCA (*r* = 0.441, *p* = 0.002 and *r* = 0.313, *p* = 0.036, respectively). There were significant mild negative correlations between quality of life (total WHOQOL-BREF score) and the number of comorbid diseases in controls (− 0.253, *p* = 0.001) but not in cases (− 0.018, *p* = 0.908).

**Table 5:** Correlations of quality of life with other psychosocial examination results and comorbidities of 209 Saudi diabetic patients by diabetic foot (DF) ulcer status

	Physical health	Psychological	Social relationships	Environment	Overall	Total (all domains)
DM with DF						
Anxiety; HAD anxiety	− 0.381**	− 0.363*	− 0.104	− 0.396**	− 0.488**	− 0.494**
Depression; HAD depression	− 0.565**	− 0.512**	− 0.212	− 0.453**	− 0.552**	− 0.624**
Somatic symptoms; PHQ-15	−0.353*	− 0.179	− 0.109	− 0.321*	−0.197	− 0.401**
Self-management; SDSCA						
Diet	−0.133	0.243	0.070	0.049	0.441**	0.065
Exercise	0.132	−0.001	0.156	0.192	0.313*	0.190
Blood sugar testing	−0.159	− 0.109	− 0.095	0.118	− 0.083	− 0.085
Foot care	0.155	0.106	−0.100	0.129	0.102	0.203
Total	−0.010	0.139	−0.021	0.189	0.334*	0.161
Number of comorbid diseases	0.096	0.047	−0.039	− 0.065	− 0.020	− 0.018
DM without DF						
Anxiety; HAD anxiety	−0.562**	− 0.547**	− 0.350**	−0.362**	− 0.365**	−0.587**
Depression; HAD depression	−0.521**	−0.488**	− 0.411**	−0.385**	− 0.341**	−0.574**
Somatic symptoms; PHQ-15	−0.654**	−0.462**	− 0.324**	−0.368**	− 0.352**	−0.582**
Self-management; SDSCA						
Diet	0.078	0.127	0.052	0.156*	0.084	0.108
Exercise	0.226**	0.005	−0.043	0.073	0.034	0.109
Blood sugar testing	−0.087	−0.049	0.101	−0.007	− 0.033	−0.064
Foot care	0.006	0.138	0.146	0.144	0.109	0.127
Total	0.036	0.080	0.059	0.111	0.057	0.068
Number of comorbid diseases	−.411**	−0.149	−0.120	−0.106	−0.086	− 0.253**
Total						
Anxiety; HAD anxiety	−0.525**	−0.509**	− 0.293**	−0.368**	− 0.387**	−0.559**
Depression; HAD depression	−0.534**	−0.491**	− 0.376**	−0.397**	− 0.382**	−0.582**
Somatic symptoms; PHQ-15	−0.575**	−0.396**	− 0.274**	−0.358**	− 0.319**	−0.532**
Self-management; SDSCA						
Diet	0.016	0.135	0.038	0.127	0.148*	0.084
Exercise	0.212**	0.009	0.006	0.094	0.092	0.137*
Blood sugar testing	−0.118	−0.065	0.050	0.024	−0.046	−0.073
Foot care	−0.002	0.099	0.085	0.136	0.087	0.111
Total	0.010	0.073	0.048	0.140*	0.095	0.079
Number of comorbid diseases	−.300**	−0.105	−0.106	− 0.092	−0.075	− 0.201**

Correlation is significant at the 0.05 level (*) or the 0.01 level (**). Abbreviations as listed in Table 3

### Multivariate analysis for quality of life

In multivariate linear regression analysis adjusting for demographic and clinical characteristics of the patients, only depression remained a significant independent determinant of quality of life. With each one point increase in the HAD score for depression, there was a decrease by 0.069 in the total WHOQOL-BREF scores of quality of life (*p* = 0.024). The association between depression and quality of life remained irrespective of the status of diabetic foot ulcers, which by itself was not a significant determinant of quality of life (unstandardized beta = 0.989, *p* = 0.848), please, see Table 4.

## Discussion

The main finding in the current study was the lack of difference in quality of life between those with and without diabetic foot ulcers. While this finding contradicts the majority of previous studies that showed lower levels of quality of life among patients with diabetic foot ulcers compared to those without diabetic foot ulcers [12–14], several important differences should be highlighted. The majority of previous studies used generic tools, such as the 36-item Short-Form Health Survey (SF-36) [12, 32], which focus on health domains with less emphasis on the cultural, psychological, and environmental context of the quality of life compared to the WHOQOL-BREF [33]. For example, in a recent meta-analysis of 12 studies that examined quality of life among patients with diabetic foot ulcers, 9 studies used SF-36 and one study used the WHOQOL-BREF [12]. Additionally, the WHOQOL-BREF measures self-perception of overall quality of life and general health, which could be influenced by expectations, standards and concerns that can be different in different cultures. While it is difficult to compare quality of life using different tools, the level of quality of life in the current study was probably higher than that seen in previous studies. For example, the average scores in the current study were > 70% for all WHOQOL-BREF domains, compared with 50 or less in most of the domains in previous studies [12, 14, 32]. This difference may be related to the better social support that the patients in both groups received within a generally conservative community characterized by the presence of extended families and religious connectedness [15]. For example, only 12.1% of our patients lived alone, close to 80% received social support from family or friends, and close to 70% reported improved family relationships after their diagnosis of diabetes. The higher levels of quality of life in both groups in the current study may have masked the difference between the two groups, if any.

Unexpectedly, the current finding showed lack of differences in anxiety, depressive symptoms, and severity of somatic symptoms between those with and without diabetic foot ulcers. This was inconsistent with studies that linked anxiety and depressive symptoms with poor quality of life among patients with diabetic foot ulcers [17, 18]. The observed lack of differences in the above psychosocial determinants between the groups of diabetic foot ulcers may explain at least partially the observed lack of difference in quality of life between the groups of diabetic foot ulcers.

Self-management is known as a core preventive measure for diabetic foot ulcers [5, 6]. Unexpectedly, patients with diabetic foot ulcers in the current study had better self-management. Moreover, patients with diabetic foot ulcers who had better self-management had also poor glycemic control. While it is difficult to explain such discrepancy, it may reflect a temporary increased willingness of patients with diabetic foot ulcers to follow self-management recommendations to treat current ulcer and prevent its recurrence [6]. Additionally, the self-reported nature of the SDSCA measure should also be considered.

Psychological factors in the current study were generally associated with poor quality of life. Similarly, several studies showed that depressive and anxiety symptoms negatively impact quality of life in patients with diabetic foot ulcers [17, 18] and those with diabetes but without diabetic foot ulcers [34, 35]. The development of depression among diabetic patients has been shown to augment the negative impact of diabetes on the physical and mental components of quality of life [35]. This augmentation may be mediated by poor adherence to medication indications, healthy lifestyle, and metabolic dysfunction [35, 36].

The severity of somatic symptoms in the current study was strongly correlated with poor quality of life in univariate analysis. While this correlation has been shown in other types of patients [37], we could not identify studies that examined the severity of somatic symptoms in relation to quality of life among patients with diabetic foot ulcers. The negative impact of somatic symptoms on quality of life may be mediated by pain and psychological symptoms [12, 37, 38]. Interestingly, the impact of pain on activity is part of the physical component of quality of life in both the WHOQOL-BREF and SF-36 [12, 30]. Similar to previous studies, diabetes self-management in the current study was associated with better quality of life [39]. Quality of life and self-management have been shown in previous studies to be positively associated among patients with [39] and without diabetic foot ulcers [40].

The current study is considered the first local study to examine quality of life and its psychosocial and related determinants in a controlled design. Additionally, the study outcomes were measured using standard validated tools. Nevertheless, a number of limitations are acknowledged. The case-control design does not prove causations but rather associations. This highlights the need for future longitudinal studies to confirm the current findings. As a two-center single-institution study, the current findings should be generalized cautiously. While the sample size had sufficient power to examine the main study objective, it may be underpowered for studying secondary objectives. The self-reported nature of the outcome tools makes it difficult to exclude recall bias. However, the current study is a valuable addition to the research on quality of life among patients with diabetes.

## Conclusion

In conclusion, quality of life was similar in patients with and without diabetic foot ulcers. Anxiety, depressive symptoms, and the severity of somatic symptoms were similar in both groups but were strongly correlated with poor quality of life in both groups. Self-management which was associated with diabetic foot ulcers had a positive but weak correlation with quality of life. Depressive symptoms were independent determinant of poor quality of life. There may be a need for interventional studies to assess the impact of preventive programs focused on depression and anxiety on different domains of quality of life.

## Data Availability

All data generated or analysed during this study are included in this published article.

## Abbreviations

HAD: The Hospital Anxiety and Depression scale
KSUMC: King Saud University Medical City
PHQ-15: The Patient Health Questionnaire Physical Symptoms
SDSCA: The Summary of Diabetes Self-Care Activities
WHOQOL-BREF: The World Health Organization’s Quality of Life Instrument-short version
